# Endobronchial Tuberculosis Mimicking Asthma

**DOI:** 10.1155/2015/781842

**Published:** 2015-12-20

**Authors:** Serap Argun Baris, Tuğba Onyilmaz, Ilknur Basyigit, Hasim Boyaci

**Affiliations:** ^1^Department of Pulmonary Diseases, Kocaeli University Faculty of Medicine, 41300 Kocaeli, Turkey; ^2^Department of Pulmonary Diseases, Mardin Government Hospital, Mardin, Turkey

## Abstract

Endobronchial tuberculosis (EBTB) is defined as tuberculosis infection of the tracheobronchial tree with microbial and histopathological evidence. The clinical symptoms of the diseases are nonspecific. Chronic cough is the major symptom of the disease. The diagnosis is often delayed due to its nonspecific presentation and misdiagnosed as bronchial asthma. This case is presented to recall the notion that the endobronchial tuberculosis can mimic asthma and the importance of bronchoscopic evaluation in a patient with chronic cough and treatment resistant asthma.

## 1. Introduction

Endobronchial tuberculosis (EBTB) is tuberculosis infection of the tracheobronchial tree with microbial and histopathological evidence. The clinical symptoms of the disease are nonspecific. The diagnosis is often delayed due to its nonspecific presentation and radiographic findings [1]. It is often misdiagnosed as bronchial asthma due to clinical symptoms [2, 3]. Furthermore, bronchoscopic findings of endobronchial tuberculosis can mimic foreign body aspiration or lung cancer [4, 5]. This case is presented for recalling the importance of bronchoscopic evaluation in patients with persistent cough and treatment resistant asthma.

## 2. Case Report

A fifty-year-old woman was admitted to outpatient clinic with the complaints of increased dyspnea and dry cough within last week. She had a history of chronic cough and dyspnea for six months. It was reported that the symptoms were intermittent at the beginning of the disease. However, the frequency of the symptoms was increased and became persistent. Also she had a chest pain of left hemithorax and loss of appetite. She was nonsmoker and her medical history was unremarkable except for asthma which was diagnosed in a secondary hospital four months ago. The medical records of this hospital showed that asthma was diagnosed according to clinical signs since she was unable to understand and perform pulmonary function test maneuvers. She stated that there was no clinical improvement despite the use of inhaler therapy. Also, she did not report any symptoms of gastroesophageal reflux and allergic rhinitis. In family history, the brother of the patient had been treated with antituberculosis drugs many years ago.

**Figure 1:**
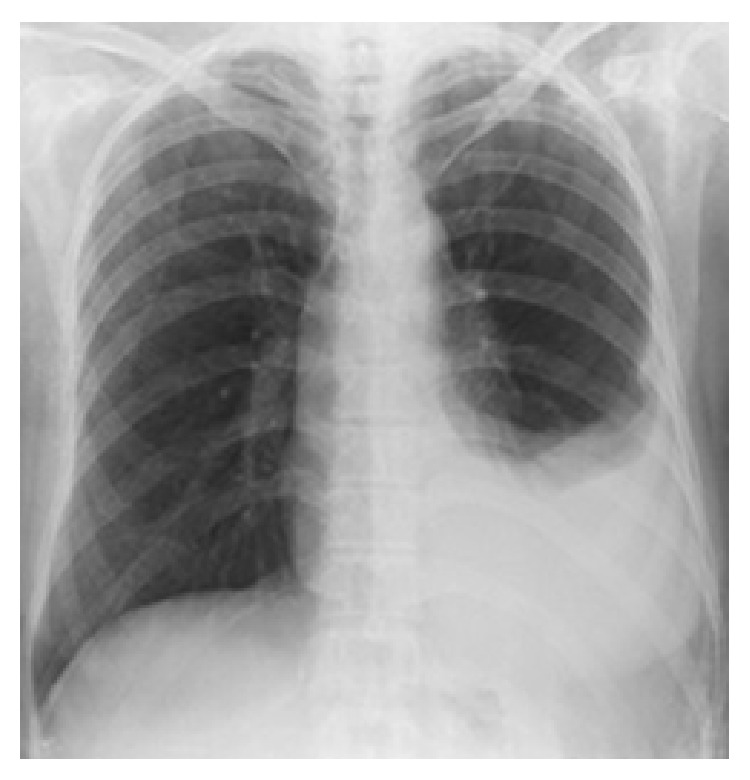
A free-flowing pleural effusion through left middle zone in chest X ray.

Vital signs of the patient were normal. Physical examination revealed dullness with percussion in left lower lobe. There was a free-flowing pleural effusion through left middle zone on chest X-ray (Figure 1). Laboratory findings were as follows: Sedimentation: 40 mm/h, WBC: 7650/mm^3^, Hb: 12.9 g/dL, Hct.: 39.7%, Plt.: 568000/mm^3^, and CRP: 0.72 mg/dL. The biochemical parameters were in normal limits. Pulmonary function test which was performed in our clinic to confirm the diagnosis of asthma was also normal.

**Figure 2:**
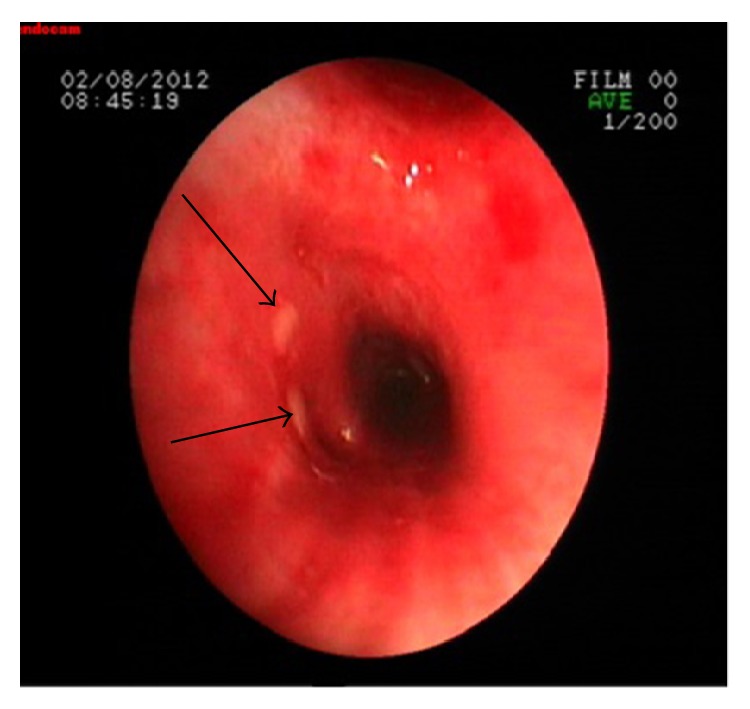
The fiberoptic bronchoscopy revealed a polypoid, necrotic endobronchial lesion in the left lower lob superior segment.

Diagnostic thoracentesis was performed. Pleural fluid was exudate. Gram and acid-fast bacilli (AFB) staining of the fluid were normal. The adenosine deaminase level of pleural fluid was 93 U/L. The fiberoptic bronchoscopy revealed a polypoid, necrotic endobronchial lesion in the left lower lob superior segment (Figure 2). Bronchial biopsy and bronchial lavage were performed. AFB staining of the bronchial lavage fluid was negative. The histopathological evaluation of the biopsy specimens revealed granulomatous inflammation, CD68(+) histiocytic cells, and CD3(+) extensive lymphocytic inflammation. Histopathologic findings were reported as tuberculosis.

Clinical, radiological, and histopathological assessments were considered to be tuberculosis. Antituberculosis treatment was started with 4 drugs including isoniazid, rifampicin, ethambutol, and pyrazinamide because of high drug resistance in Turkey. The bronchial lavage Lowenstein Jensen medium culture was positive on the 26th day of the bronchoscopy and there was no drug resistance. Patient received six months of tuberculosis treatment. The clinic and radiologic findings improved after the treatment.

## 3. Discussion

Endobronchial tuberculosis (EBTB) is defined as tuberculosis infection of the tracheobronchial tree with microbial and histopathological evidence. It is seen in 10–40% of patients with active pulmonary tuberculosis [6]. The pathogenesis of EBTB is not well known. It is suggested that five potential mechanisms are responsible for the development of the disease: (I) direct invasion from an adjacent parenchymal focus; (II) implantation of the organisms from infected sputum; (III) hematogenous spread; (IV) erosion of a lymph node inside a bronchus; (V) lymphatic drainage from the parenchyma towards the peribronchial region [7].

Clinical features of the EBTB are nonspecific and they differ between various types and stages of endobronchial tuberculosis [8]. Common symptoms are cough, hemoptysis, sputum production, wheezing, chest pain, fever, and dyspnea [8, 9]. Bronchial hyperreactivity can be seen in a considerable number of patients with EBTB. Also EBTB can be misdiagnosed as asthma [2, 3]. So the differential diagnosis of EBTB and cough variant asthma has a great importance. Endobronchial tuberculosis is difficult to diagnose, because the lesion is not evident in the chest radiograph [9]. Ten to 20 percent have normal chest radiograph. Therefore, a clear chest radiograph does not exclude the diagnosis of EBTB [6]. Our patient also had chronic cough and diagnosis of asthma for six months. She had two chest radiographies which were taken during the follow-up for asthma. Although these chest radiograms were normal, chest X-ray taken at the moment of admission to our clinic revealed left pleural effusion.

In the absence of parenchymal disease, endobronchial tuberculosis is less well-recognized and can lead to difficulties in diagnosis [10]. Persistent symptoms, history of household contact of tuberculosis, and developing new radiologic abnormality have suggested misdiagnosis of asthma in our case.

Bronchoscopy and computed tomography are the methods of diagnosis of bronchial involvement [6]. The diagnosis and follow-up of EBTB lesion during treatment mainly depend on bronchoscopy [4]. Bronchoscopic sampling has been the key to the diagnosis producing more than 90% yield on smear as well as on culture. Endobronchial tuberculosis is classified into seven subtypes: actively caseating, fibrostenotic, oedematous-hyperaemic, tumorous, ulcerative, granular, and nonspecific bronchitic type according to bronchoscopic features [4]. The gold standard of the EBTB diagnosis is microbial and histopathological evidence of disease. Diagnostic fiberoptic bronchoscopy was also performed and it was found that there was a polypoid necrotic endobronchial lesion in our case. EBTB is proved by both microbial and histopathological specimens.

The most important goal of treatment in active endobronchial tuberculosis is the eradication of tuberculosis bacilli [9]. Early diagnosis and effective treatment of endobronchial tuberculosis with antituberculosis drugs have a great importance for completing therapy without complications such as severe bronchostenosis [10]. Antituberculosis treatment was given to our patient for six months. Control bronchoscopy could not be performed because the patient did not agree. The clinical and radiologic findings of the patient were regressed after the treatment.

## 4. Conclusion

Endobronchial tuberculosis can mimic asthma due to nonspecific clinical specialties and a normal chest radiograph does not exclude the diagnosis. It is suggested that bronchoscopy should be performed for the differential diagnosis of chronic cough and drug resistant asthma. Furthermore, tuberculosis should be kept in mind in patients with household contacts and living in endemic regions of tuberculosis.
